# Screening for Resistance to PVY in Australian Potato Germplasm

**DOI:** 10.3390/genes11040429

**Published:** 2020-04-16

**Authors:** Anthony T. Slater, Lee Schultz, Maria Lombardi, Brendan C. Rodoni, Chris Bottcher, Noel O. I. Cogan, John W. Forster

**Affiliations:** 1 Agriculture Victoria, AgriBio, 5 Ring Road, La Trobe University, Bundoora, VIC 3083, Australia; 2 School of Applied Systems Biology, La Trobe University, Bundoora, VIC 3086, Australia

**Keywords:** Potato virus Y, potato breeding, marker-assisted selection, phenotype, resistance

## Abstract

Potatoes are an important human food crop, but have a number of yield limiting factors, including disease susceptibility. *Potato virus Y* (PVY) is found worldwide, and is one of the main virus problems for potato growers. PVY is transmitted by aphids and mechanically by machinery, tools and people, and symptoms are variable across cultivars and strains, including being symptomless in some cultivars. Therefore, breeding resistant cultivars is the best way to control this virus. This study phenotypically screened 74 of the main commercial cultivars and a few other select cultivars grown in Australia, in order to identify sources of resistance to PVY. The cultivars were screened against PVY^O^ and PVY^NTN^, with 23 out of 71 resistant to PVY^O^ and 13 out of 74 resistant to PVY^NTN^, and all these 13 were resistant to both strains. When the phenotypic screening was compared to the results listed on the European Cultivated Potato Database, the majority of results were found to be consistent. We then evaluated three molecular markers RYSC3, M45, and STM0003 for the extreme resistance genes *Ry_adg_* and *Ry_sto_,* to validate the usefulness of the markers for marker-assisted selection (MAS) on Australian germplasm. The degree of correlation between the resistance phenotypes and the RYSC3, M45, and STM0003 markers for *Ry_adg_* and *Ry_sto_* conferred PVY resistance was determined. Three cultivars amplified the RYSC3 marker, while the M45 marker amplified the same 3 and an additional 9. Of the 12 cultivars, 11 phenotyped as resistant, but 1 was susceptible. The STM0003 marker was amplified from only 2 cultivars that both had resistant phenotypes. The RYSC3, M45, and STM0003 markers were therefore able to identify all the 13 cultivars that were resistant to both strains of PVY. Therefore, these markers will enable the identification of genotypes with resistance to PVY, and enable PVY resistant parents to be used for the development of superior progeny; these genetic markers can be used for MAS in the Australian potato breeding program.

## 1. Introduction

Potatoes are an important human food crop produced globally, but it has a number of yield limiting factors, including disease susceptibility, such as *Potato virus Y* (PVY). PVY is a potyvirus that is found worldwide and is one of the main virus problems for seed and commercial potato growers [1,2,3,4]. PVY is transmitted by over 50 species of aphids [5], and as it is transmitted in a non-persistent manner, the virus can be acquired by the aphid and transmitted to a new plant as soon as feeding begins. As well as being transmitted by aphids, PVY can also be transmitted mechanically by machinery, tools, and by brushing plants while walking through the field. Symptom expression is cultivar and viral strain dependent, but can include vein necrosis, mottling, yellowing of leaflets, leaf-dropping, plant dwarfing, and premature plant death [1,6]. Yield losses may be as high as 80% [1,7]. There are also cultivars, such as Shepody, for which PVY may be asymptomatic rather than producing the typical mosaic symptoms on the leaves, and this can create a major risk for the spread of the virus in certified seed schemes [8]. Furthermore, the lack of symptoms in infected plants means that visual inspection will generally be ineffective, and that such plants provide a source of PVY inoculum in production areas. Therefore, breeding resistant cultivars is the best way to control this virus and remove inoculum.

An effective potato breeding program aims to breed new cultivars that have traits that are superior or equivalent to those of current cultivars. In order to do this, potential parents need to be identified, which contain genes that control at least 40 desirable traits [9,10]. A list of traits that are important for the breeding of improved cultivars in Australia and their genetic control were listed by Slater et al. [10]. A number of these characters are considered to be controlled by single dominant genes, as they exhibit qualitative phenotypic variation. Simko et al. [11] listed 39 major R-genes for disease resistance in potato. These include major resistance genes for potato cyst nematode, PVY, *Potato virus X* (PVX), *Potato virus S* (PVS), root knot nematode, and late blight. Major genes have also been mapped for the control of morphological traits such as flesh, skin and flower colour, tuber shape and eye depth [12,13], and tuber physiology and quality traits [13].

Understanding the genetic control of the host-pathogen resistance interaction is important in developing resistance cultivars. There are two types of single dominant resistance genes for PVY, namely hypersensitive response or *Ny* genes that are effective against individual strains of PVY, and extreme resistance or *Ry* genes that are effective against all strains. The hypersensitive response results in cell death, and inhibits virus spread from cell to cell and through the vascular system, while extreme resistance is characterised by a strong suppression of virus replication [4,14,15].

The PVY extreme resistance genes that have been identified are *Ry_adg_*, *Ry_sto_*, and *Ry_chc_* [2,16]. European potato breeding programs have used *Ry_sto_* [17,18], while *Ry_adg_* is an important resistance gene that is used in North America [6,19]. As the majority of potato germplasm in Australia was introduced from Britain, Europe, and North America, the extreme *Ry* genes *Ry_sto_* and *Ry_adg_* were of most interest as they confer resistance against all strains of PVY. A recent PVY isolate sequencing study has also identified that PVY^O^, PVY^C^, PVY^N^, and PVY^NTN^ occur within Australia (Zheng, pers. comm), so resistance against all these strains is important.

In order to identify potential resistant parents, the desirable genotypes must be characterised on the basis of their phenotype. This is not a trivial matter, as the results must be reproducible within the field conditions in which the cultivars will be commercially grown. Field trials are valuable, but can be expensive and time-consuming, while screening results may be vulnerable to environmental variation caused by factors such as disease pressure. Laboratory-based assays can be conducted on plants, which have the capacity to detect the presence of the pathogen in susceptible plants, whether symptoms are exhibited, or infected plants were asymptomatic and may be regarded as tolerant. Greenhouse-based screening can provide an attractive method for plant evaluation [11], and would be quite suitable for screening for resistance to PVY.

DNA-based genetic markers also provide great potential to assist plant breeders in the identification of genes of interest for the development of new cultivars. Marker-assisted selection (MAS) could be utilised in a potato breeding program based in Australia for traits of interest, when they are more cost-effective than conventional screening or when they can provide an earlier, or more effective screening methodology, and MAS has been shown to be more cost-effective than virus glasshouse screening trials [20]. The initial traits targeted for the development of MAS need to be relevant to Australian potato breeding priorities, and broadly applicable to Australian germplasm; PVY resistance is a high priority.

The resistance gene, *Ry_sto_*, was mapped to a 0.6 cM interval between two flanking markers, M17 and M6, on chromosome 11 [21], and this region also contained the marker M45, but the accuracy of the pedigree information used in this study has been questioned [2,22,23]. In contrast to the results of Brigneti et al. [21], Song et al. [23] found that *Ry_sto_* was on chromosome 12 located to an interval between the marker loci GP268 and TG28, and demonstrated co-segregation with seven amplified fragment length polymorphism (AFLP) markers and the simple sequence repeat (SSR) marker STM0003_111_. Song and Schwarzfischer [15] noted that a Hungarian breeding program had located *Ry_sto_* 2.1 cM from STM0003_111_ in a population derived from the cultivar White Lady. This study also analysed the pedigrees of European cultivars which exhibit extreme resistance to PVY that have the *Ry_sto_* indicative markers, and found that they all descended from *Solanum stoloniferum* [15]. Valkonen et al. [18] repeated the screening process on the population generated by Flis et al. [22], and mapped all the markers that co-segregated with *Ry_sto_* [22,23,24], with similar results. However, screening of a diploid population from a third *Ry_sto_* source, with cleaved amplified polymorphic sequence (CAPS) markers developed from GP122 and the SSR marker STM0003, located the relevant loci to 15.2 cM from *Ry_sto_*. These authors concluded that the *Ry_sto_* gene from all three sources mapped to slightly different locations, in a region distal to GP122 towards the end of chromosome 12, and that these markers could be genotype specific [18]. Cernák et al. [25] located STM0003_111_ 2.95 cM from *Ry_sto_* in the resistant cultivar White Lady, making STM0003 a good marker for MAS. Further mapping has located *Ry_sto_* on the DMB114 superscaffold of the DM genome [26], and to the single candidate gene, *c630* [27].

The *Ry_adg_* gene was mapped to the proximal region of chromosome 11, with the nearest marker, TG508, located at a maximum distance of 2.0 cM [28]. Further analysis identified TG508 1.3 cM from *Ry_adg_*, and in close linkage to six other markers, including ADG2 in a diploid mapping population [29]. ADG2 was then used to develop a CAPS marker [30], and then two sequence characterised amplified region (SCAR) markers, RYSC3 and RYSC4 [31] predictive for *Ry_adg_*. RYSC3 was identified in 14 resistant genotypes with *Ry_adg_*, and was absent from all the susceptible genotypes, while RYSC4 only matched in 96.1% of the population. Whitworth et al. [19] used RYSC3, RYSC4, and ADG2 to confirm the presence of *Ry_adg_* in resistant cultivars when challenged with the viral strains PVY^O^, PVY^N^, and PVY^N:O^. Ottoman et al. [6] screened breeding populations with the markers RYSC3 and ADG2 and identified a 3.6% disagreement between the marker result and the phenotypic score. Dalla Rizza et al. [32] used M45 and RYSC3 to screen for the presence of both *Ry_sto_* and *Ry_adg_*, but obtained near identical results. Valkonen et al. [18] noted these results, as well as their own screening results, and concluded that the gene mapped by Brigneti et al. [21] was actually *Ry_adg_*. Brigneti et al. [21] reported from a high-resolution mapping study that the markers M45 and M5 co-segregated with the *Ry* gene and were 0.3 cM from M6 on one side and M17 on the other, while Jara Vidalon (2010) in Herrera et al. [33] estimated that M45/M5 was 0.05 cM from M6 and 0.2 cM from RYSC3 from a large mapping population of 6521 individuals. Therefore, M45 and RYSC3 are good markers for MAS.

The aim of this study was to phenotype the Australian parental collection and select cultivars for PVY resistance and then validate the effectiveness of the RYSC3, M45, and STM0003 markers for *Ry_adg_* or *Ry_sto_* conferred PVY resistance, in order to use them in MAS in the Australian potato breeding program.

## 2. Materials and Methods

### 2.1. Plant Material

Plant materials used in this study were taken from tetraploid potato cultivars and breeding lines selected from the germplasm collection of the Australian potato breeding program. All planting material for phenotypic trials consisted of small tubers produced by the breeding program during the previous summer. Leaf material for DNA extraction was collected from plants growing in the annual parental material multiplication trial of the breeding program. The leaf samples consisted of approximately 1.5 cm^2^ of leaf material placed directly into 96 well plates, with a duplicate sample placed in a resealable plastic bag for storage at −70 °C.

### 2.2. Potato Virus Y Phenotyping Assays

Separate trials were run to screen for PVY^O^ and PVY^NTN^ resistance, although the same phenotyping protocol was used. In total, there were 10 trials for PVY^O^ and PVY^NTN^ resistance during 2010, 2011, and 2012.

Selected potato cultivars were sown in either 150 mm or 180 mm pots, depending on the trial. Pots were two-thirds filled with a pine-based potting mix. One small tuber or cut tuber from each cultivar was then partially embedded in the potting mix, and the pots were topped up with additional potting mix, as required. Pots were placed on raised benches in a glasshouse at 22–28 °C (day) and 14–20 °C (night), with ambient lighting and humidity, and were watered by hand. The tubers were fertilised initially with 10 g of Osmocote Plus. Known resistant and susceptible cultivars were included in the trials as positive and negative controls.

Four plants of each cultivar were separately inoculated with either PVY^O^ or PVY^NTN^. Leaves from infected PVY^O^ or PVY^NTN^ potato or tobacco plants were ground in chilled extraction buffer, at the rate of approximately 1 in 10 dilution of leaf material to the buffer, to create the inoculum. The extraction buffer was prepared by adding 7.5 g NaH_2_PO_4_2H_2_O (0.05 M phosphate buffer) with 1% (*w/v*) Na_2_SO_3_ to 1 L distilled water, and the pH adjusted to 7.4. Plant leaves identified for inoculation were powdered with carborundum powder, and the PVY^O^ or PVY^NTN^ inoculum was then rubbed across the leaves. The carborundum was used to graze the leaf surface to facilitate virus transmission into the leaf. Two plants of each cultivar were also inoculated with buffer containing uninfected potato leaf material, and two plants were left uninoculated. Plants were tested for virus infection using enzyme-linked immunosorbent assay (ELISA) by taking leaf samples after 30 days, and then cultivars with negative results were re-tested after 40 days. Young fully expanded leaves were sampled from the growing tip, to avoid collection of inoculated leaves that could contain a trace of the virus from the inoculation. Cultivars were considered susceptible if virus was detected, but were considered to be resistant if no virus was detected over three independent trials.

### 2.3. Enzyme-Linked ImmunoSorbent Assay Testing for Virus

A stock solution (5×) of Phosphate Buffered Saline (5× PBS) was prepared by combining 200 g NaCl, 5.0 g KH_2_PO_4_, 28.8 g HNa_2_O_4_P, and 5.0 g KCl, and was made up to 5 L with distilled water. The pH of the 5× PBS stock solution was adjusted to pH 7.4. From the stock solution, PBS Tween 1× was prepared by combining 1 L 5× PBS, 4 L distilled water and 2.5 mL Tween 20. Leaf samples (approximately 1 g) were crushed between two rollers in a motorised leaf press. Ten mL of extraction buffer (1 L PBS Tween 1×, 20.0 g polyvinylpyrrolidine (PVP) and 4.0 g skim milk powder) was poured down the rollers and the run off, comprising sap and extraction buffer, was collected in a 30 mL sample cup. Rollers were washed with tap water and wiped clean between samples to prevent cross-contamination of samples.

Antiserum for PVY was obtained from Agdia, Inc. (New Town, Tasmania, Australia), and a double antibody sandwich ELISA technique was used [34]. The antiserum (100 μL/well) was pipetted into 96 well microtitre plates (Nunc) at 1/200 dilution in carbonate coating buffer (1.59 g Na_2_CO_3_ and 2.93 g NaHCO_3_, and made up to 1 L with distilled water and adjusted to pH 9.6) as stated in the supplier’s instructions for each ELISA kit, followed by incubation at 37 °C for 4 h or at 4 °C overnight, and subsequent washing. Microtitre plates were washed between each step by filling of each well with wash buffer (100 mL PBS 10× stock, 900 mL distilled water, 0.5 mL Tween 20, and 1.0 g milk powder, made up to 1 L and adjusted to pH 7.4) and soaking for at least 3 min. This step was repeated three times. After the final wash, plates were emptied of wash buffer, and allowed to drain upside-down over paper towels for approximately 5 min before the next step.

Samples, including negative and positive controls were added to 96 well microtitre plates in adjacent duplicate wells. The plates containing sample extracts were incubated overnight at 4 °C, as recommended by the manufacturer’s instructions. Antiserum conjugated with alkaline phosphatase was prepared at the same dilution that was used to coat the plate. Conjugated antiserum was diluted in conjugation buffer (100 mL PBS 10×, 2.0 g bovine serum albumin, 20.0 g PVP, made up to 1 L with distilled water and adjusted to pH 7.4). An aliquot of 100 μL dilute conjugated antiserum was added to each well, and the microtitre plates were incubated at 37 °C for 4 h. Plates were washed as described above. Substrate tablets, each containing 5 mg p-nitrophenyl phosphate, were added at a rate of 1 tablet per 10 mL of substrate buffer (48.5 mL diethanolamine and 400 mL of distilled water adjusted to pH 9.8), and 100 μL of substrate buffer was added to each well. Plates were incubated for 30–60 min at room temperature in order to allow colour development, and absorbance of each well was read at 405 nm using a Titertek photometer (Flow Laboratories, Helsinki, Finland). Samples were considered positive if the optical density reading was above 0.160, which equates to approximately 2.5 times the optical density of the negative control, and negative if below 0.120. Readings between these values were considered ambiguous, and the cultivar was re-tested.

### 2.4. DNA Isolation

Total genomic DNA was extracted from c. 1.5 cm^2^ frozen leaf material using the DNeasy 96 Plant Kit (Qiagen, Hilden, Germany) following modified manufacturer’s instructions. Briefly, leaf material was ground to a powder using a MM300 Mixer Mill (Retsch, Haan, Germany), to which the AP1 lysis buffer containing RNAse A and Reagent DX was added and homogenised with the Mixer Mill. Buffer AP2 was added to the homogenised leaf/AP1 mixture and incubated at −20 °C for 10 min. Samples were then centrifuged at 5800 g for 5 min. For a high-throughput, automated approach, the remainder of the extraction protocol was carried out using a Biomek FX robot (Beckman-Coulter, Brea, CA, USA) with a customised program, with the extraction being performed following the manufacturer’s instructions. DNA quality was assessed using a 1% (*w/v*) agarose gel in 0.5 × TBE with DNA ladder, Hyperladder I (Bioline, London, UK).

### 2.5. PVY Marker Assays

#### 2.5.1. RYSC3 Marker Assay

The SCAR marker RYSC3 has been shown to produce a 321 bp band in the presence of the PVY resistance gene *Ry_adg_*. Forward (5′-ATACACTCATCTAAATTTGATGG-3′) and reverse (5′-AGGATATACGGCATCATTTTTCCG A-3′) RYSC3 primers were designed by Kasai et al. [31] with the forward primer labelled with the fluorochrome FAM for detection following capillary electrophoresis. PCR reactions were carried out in a total volume of 10 μL containing 20 ng of template DNA, 1 × ImmoBuffer (Bioline, London, UK), 0.25 mM dNTPs (Bioline, London, UK), 0.25 µM of each primer, and 0.5 Unit Immolase™ DNA Polymerase (Bioline, London, UK). Cycling conditions for PCR were: 94 °C for 10 min followed by 30 cycles of 94 °C for 45 s, 60 °C for 45 s, 72 °C for 45 s, and a final step of 72 °C for 10 min. The amplified products were prepared for resolution on an ABi3730xl DNA Analyzer (Applied Biosystems, Foster City, CA, USA) and analysed with GeneMapper V3.7 (Applied Biosystems, Foster City, CA, USA), as described in Schultz et al. [35]. The presence or absence of the 321 bp fragment was then compared to the phenotypic result for each genotype in order to validate the effectiveness of the marker.

#### 2.5.2. M45 Marker Assay

The M45 marker with forward (5′-GACTGCGTACATGCAGCT-3′) and reverse (5′-GATGAGTCCTGAGTAAGGA-3′) primer sequences were developed by Brigneti et al. [21]. The M45 marker is linked to the *Ry_adg_* gene [18]. PCR reaction conditions for amplification of the M45 marker were similar to those used for RYSC3, with minor modifications. PCRs were carried out in a total volume of 10 μL containing 20 ng of template DNA, 1 × ImmoBuffer (Bioline, London, UK), 0.25 mM dNTPs (Bioline, London, UK), 0.25 µM of each primer, and 0.5 Unit Immolase™ DNA Polymerase (Bioline, London, UK). Cycling conditions for PCR were: 94 °C for 10 min, followed by 35 cycles of 94 °C for 45 s, 60 °C for 45 s, 72 °C for 45 s, and a final step of 72 °C for 10 min. The forward primer was fluorochrome-labelled with HEX for capillary electrophoresis as described in Schultz et al. [35]. Cultivars that amplified a 493 bp fragment are *Ry_adg_* positive. The 116 bp band that is present in all lanes is an internal control for DNA quality. The presence or absence of the 493 bp fragment was then compared to the phenotypic result for each genotype to validate the effectiveness of the marker.

#### 2.5.3. STM0003 Marker Assay

The SSR marker STM0003 [36] has been shown to detect a 111 bp allele diagnostic for potato cultivars that express extreme resistance to PVY and carry *Ry_sto_* [23]. The forward (5′-GGAGAATCATAACAACCAG-3′) primer was fluorochrome labelled with HEX for detection following capillary electrophoresis with the reverse (5′-AATTGTAACTCTGTGTGTGTG3′) primer, as described by Milbourne et al. [36]. PCR reaction conditions for amplification and analysis of the STM0003 marker were similar to those used for RYSC3, with minor modifications. PCRs were carried out in a total volume of 10 μL containing 20 ng of template DNA, 1x ImmoBuffer (Bioline, London, UK), 0.25 mM dNTPs (Bioline, London, UK), 0.25 µM of each primer, and 0.5 Unit Immolase™ DNA Polymerase (Bioline, London, UK). Cycling conditions for PCR were: 94 °C for 10 min followed by 30 cycles of 94 °C for 45 s, 58 °C for 45 s, 72 °C for 45 s, and a final step of 72 °C for 10 min. The amplified products were prepared for resolution on an ABi3730xl DNA Analyzer (Applied Biosystems, Foster City, CA, USA) and analysed with GeneMapper V3.7 (Applied Biosystems, Foster City, CA, USA). The presence or absence of the 111 bp fragment was then compared to the phenotypic result for each genotype in order to validate the effectiveness of the marker.

## 3. Results

### 3.1. PVY Resistance Screening

**Figure 1:**
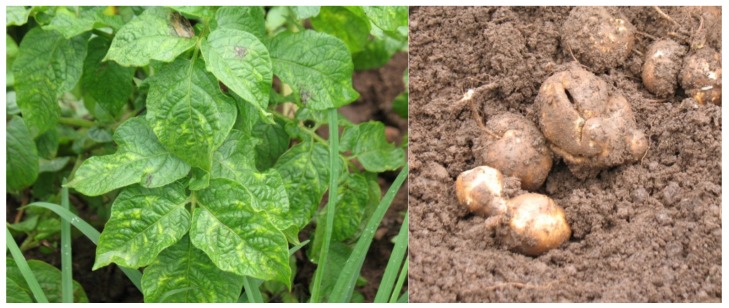
Symptoms of Potato Virus Y infection on the cultivar Atlantic. Note the leaf mottling and the tuber necrosis.

The majority of infected plants did not exhibit symptoms in the glasshouse, although there was slight to severe mottling on some leaves, and leaf curl, leaf stunting, necrotic spots and terminal leaf death on some plants. Leaf mottling and tuber necrosis from field plants are illustrated in Figure 1. The symptoms were occasionally, but often not, consistent across all four plants of each cultivar being tested.

The PVY^O^ screening trials demonstrated that of the 71 cultivars tested, 48 tested positive with the ELISA and are therefore susceptible, while 23 returned a negative test result on three or more occasions, and were therefore considered resistant to PVY^O^. These cultivars were Almera, BC0894-2, Carlingford, Coliban, Eos, Eva, Exton, Friar, Galil, Kestral, KT3, Lady Christl, Melody, Nadine, PO3, Red Rascal, Rioja, Royal Blue, Sequoia, Simcoe, Snowgem, White Lady, and Wontscab (Table 1).

The PVY^NTN^ screening trials demonstrated that of the 74 cultivars tested, 61 tested positive with the ELISA and are therefore susceptible, while only 13 returned a negative test result on three or more occasions, and were therefore considered resistant to PVY^NTN^. These cultivars were BC0894-2, Carlingford, Eos, Eva, Friar, Galil, KT3, Lady Christl, Melody, PO3, Rioja, Royal Blue, and White Lady (Table 1).

When the results from the PVY^O^ and PVY^NTN^ trials were compared, all 13 cultivars that were resistant to PVY^NTN^ were also resistant to PVY^O^, and 10 cultivars that were resistant to PVY^O^ were susceptible to PVY^NTN^. (Table 1). These 10 cultivars were Almera, Coliban, Exton, Kestral, Nadine, Red Rascal, Sequoia, Simcoe, Snowgem, and Wontscab (Table 1).

When the phenotypic screening was compared to the results listed on the European Cultivated Potato Database (ECPD), the majority of results were found to be consistent, although the results for Charlotte, Crystal, Denali, Desiree, Innovator, Kennebec, Sebago, and Spunta were inconsistent, where they were susceptible in our trials but listed as having moderate to high resistance on the ECPD (Table 2). It is also worth noting that the ratings for Atlantic and Nicola on the ECPD are different in the two categories, being low in one and high in the other (Table 2).

### 3.2. PVY Marker Evaluation

Seventy-four cultivars that have been phenotyped for PVY resistance were also genotyped with the markers RYSC3, M45, and STM0003. Only three cultivars amplified the RYSC3 marker, while the M45 marker was amplified by the same three cultivars and nine additional cultivars (Table 1). The three cultivars that amplified both markers were Emma, Eva, and PO3, while M45 was also amplified in BC0894-2, Carlingford, Eos, Friar, Galil, KT3, Lady Christl, Melody, and Royal Blue. These twelve cultivars were all phenotyped as resistant to both strains of PVY, except for Emma, which was identified as susceptible to both strains of PVY (Table 1). The STM0003 marker was identified in two different cultivars, namely Rioja and White Lady, which were both phenotyped as resistant cultivars. All other cultivars that did not amplify any of the markers displayed a susceptible phenotype to at least the PVY^NTN^ strain (Table 1). The cultivars that were resistant to PVY^O^ and susceptible to PVY^NTN^ did not display the marker for either extreme resistance gene, and therefore may contain a hypersensitive resistance gene.

## 4. Discussion

This study has conducted phenotypic and molecular marker screening on the main commercial cultivars and a few other select cultivars grown in Australia, in order to identify sources of resistance to PVY and validate the usefulness of three markers for MAS on Australian germplasm. The degree of correlation between the resistance phenotypes and the RYSC3, M45, and STM0003 markers for *Ry_adg_* and *Ry_sto_* conferred PVY resistance was determined. This process will enable the identification of cultivars with resistance to be used as parents for the development of superior progeny and identify effective genetic markers to enable their use in MAS in the Australian potato breeding program.

The phenotypic screening has identified the resistance status of the main commercial potato cultivars that are grown in Australia, as well as a few select cultivars, to two strains of PVY, namely PVY^O^ and PVY^NTN^, found in Australia. A total of 23 of 71 cultivars were found to be resistant to PVY^O^, while 13 of 74 were found to be resistant to both the PVY^O^ and PVY^NTN^ strains. Resistance to PVY has been attributed to three extreme resistance genes (*Ry_adg_*, *Ry_sto_* and *Ry_chc_*), which provide resistance to all strains of PVY [11], while hypersensitive resistance genes provide resistance against individual PVY strains, such as *Ny* for PVY^O^ [3,37]. Consequently, the 13 cultivars that were found to be resistant to both strains of PVY are likely to possess one of the *Ry* extreme resistance genes. It was likely that at least *Ry_sto_* was present, as it has been previously found in the cultivars White Lady and Rioja [15,25,38], which were both found to be phenotypically resistant here.

The 10 cultivars that were found to be resistant to PVY^O^ but susceptible to PVY^NTN^ are likely to possess the hypersensitive gene *Ny*, as they displayed resistance to PVY^O^. However, it is possible that the negative result could be due to a failure to inoculate any of the four replicate plants in the three screening trials. The larger number of cultivars that are resistant to PVY^O^ in comparison to PVY^NTN^ also indicates the activity of more than one resistance gene in the range of cultivars grown in Australia.

The phenotypic scores for cultivars from this study are generally consistent with the results reported from other studies. The cultivar White Lady was also reported to be resistant to PVY in four previous studies [15,25,32,38], and Cernák et al. [25] also reported Rioja to be resistant. A number of cultivars, including Atlantic [31,39,40], Bliss [37], King Edward [31,39], Kipfler [37], Pike [31], Ranger Russet [19], Russet Burbank [19,31], Shepody [8] and Umatilla [19], were reported to be susceptible elsewhere. Three cultivars have been reported to exhibit hypersensitive resistance although they tested as susceptible in this study. These cultivars were Desiree [15,39,41], Nicola [22] and Sebago [28]. Desiree has been reported to contain the *Ny_tbr_* hypersensitivity gene, which is active against PVY^O^ [39,41], and to display hypersensitive reactions of severe necrosis, leaf drop, and stunting [39]. However, Desiree was also described as displaying non-hypersensitive symptoms of severe mosaic and leaf deformation [39], and Desiree has been noted as susceptible in another study that used ELISA to detect the virus [38]. This may indicate that in Desiree, the hypersensitive reaction is not preventing some multiplication and systemic movement of the virus. This systemic virus movement may be a result of environmental influences, or of the physiology of the host plant [4].

Resistance ratings are provided for cultivars on the ECPD, but these ratings need to be considered carefully before introducing new cultivars for PVY resistance into Australia. A number of discrepancies were identified between the results of this study and the ratings on the ECPD, with the database listing eight cultivars with resistance, while they were found to be susceptible here. The difference in two of these instances (Desiree and Sebago) may be explained by failure of the hypersensitive reaction to prevent all virus movement, and the ELISA-based detection of this movement. The different ratings obtained for Nicola when challenged with the two strains may again be explained by the presence of the hypersensitive resistance gene, but the ratings for Atlantic cannot. The different ratings may also indicate that different strains of PVY are being used for phenotyping trials in various countries. Therefore, information in this database should be treated with caution until the cultivars can undergo reliable local evaluation.

PVY^NTN^ was first detected in Australia in 2003, and since then there has been a continual increase in the detection of PVY in seed crops. This observation can in part be explained by the resistance of the main commercial cultivars. Prior to 2003, the main fresh market cultivars were Coliban, Sebago, Nadine, and the red-skinned Desiree. Coliban and Nadine both tested resistant to PVY^O^ and susceptible to PVY^NTN^, and therefore are likely to possess the hypersensitive gene *Ny*. Although Desiree and Sebago both tested susceptible, these cultivars have also been reported to possess this hypersensitive resistance gene for PVY^O^ [28,39]. At that time, the main processing cultivars, Altantic and Russet Burbank, were susceptible to PVY^O^. Therefore, prior to 2003, PVY^O^ was controlled by the hypersensitive resistance gene *Ny* in the main fresh market cultivars, but following the introduction of PVY^NTN^, these cultivars lost the protection provided by *Ny*. Consequently, control of PVY must rely on strict hygiene protocols until cultivars can be bred that are resistant to all four strains of PVY that are now present in Australia.

The presence of the three markers RYSC3, M45, and STM0003 was shown to be correlated with resistance to PVY^O^ and PVY^NTN^ of the main potato cultivars grown in Australia. These are the markers that are most commonly used to predict the presence of the extreme resistance genes *Ry_adg_* and *Ry_sto_* [6,15,18,19,21,23,25,31,32,38]. In 73 of 74 cultivars, the presence or absence of one of these markers correlated with their resistance status to both virus strains. Only for the cultivar Emma did the presence of a marker disagree with a susceptible phenotypic result. Consequently, these markers are highly predictive of the PVY phenotype for the main Australian germplasm. The cultivars that were phenotypically assessed as resistant to PVY^O^ and susceptible to PVY^NTN^ are likely to possess the hypersensitive gene *Ny*, and were not expected to show a positive reaction for markers that tag these extreme resistance genes. These markers have also been shown to be highly predictive in other studies, with STM0003 mapped to 2 cM from *Ry_sto_* [15,25], RYSC3 3 cM from *Ry_adg_* [6], while M45 showed complete concordance with the phenotype in the study by Brigneti et al. [21].

There have been conflicting reports as to which extreme resistance gene is detected by the marker M45, which was first reported to detect *Ry_sto_* [21], but has been argued to actually detect *Ry_adg_* [18]. The results from this study have shown that RYSC3 and M45 were both detected in three cultivars, but no cultivars displayed positive results for both M45 and STM0003. M45 was also not detected in the cultivar White Lady, which has been reported several times to possess *Ry_sto_* [15,25,38]. Therefore, these results support the conclusion that M45 detects *Ry_adg_* rather than *Ry_sto_*.

In this study, M45 and RYSC3 were both detected in three cultivars, while M45 was then found to be present in another nine cultivars. RYSC3 and M45 were also used in another study on another set of cultivars where the results overlapped nearly perfectly, as only one resistant cultivar displayed M45 and not RYSC3 [32]. In neither study were any resistant cultivars described as possessing RYSC3 but not M45. From these observations, M45 may be closer to *Ry_adg_* if they are located on the same side of the gene, or M45 may flank *Ry_adg_* on the opposite side to RYSC3. Whichever side of the gene is flanked, M45 appears to represent a more closely linked marker than RYSC3. Ottoman et al. [6] reported that RYSC3 was 3.6 cM from *Ry_adg_*, while Brigneti et al. [21] reported that M45 co-segregated with *Ry_adg_*; this also indicates that M45 is closer to *Ry_adg_* than RYSC3. Despite Brigneti et al. [21] reporting the co-segregation of M45 with *Ry_adg_*, the linkage may still be reversed, as shown by the loss of association with the phenotype in the cultivar Emma. This loss of association with both markers in Emma is also likely to be from a single recombination event and indicate that M45 and RYSC3 are on the same side of *Ry_adg_.*

The detection of the three markers RYSC3, M45, and STM0003 in correlation with the resistant phenotype indicates that two extreme resistance genes, *Ry_adg_* and *Ry_sto_*, occur in the Australian germplasm collection, and can be used in a crossing program to develop elite resistant cultivars. The presence of the hypersensitive gene, *Ny*, may also be used, but as it would not provide resistance against PVY^NTN^, it would not be as desirable, especially as this strain causes tuber necrosis. A disadvantage of using *Ry_sto_* is that this gene is known to be linked to male sterility [15,17], and therefore could only be used as a female parent.

Potato is affected by a large number of pathogens, and breeding for resistance to these pathogens is critical for the development of new, superior cultivars. Resistance to pathogens in plants has been attributed to receptors in non-mobile cells, unlike the mobile resistance mechanisms that are characteristic in animals. Plant pathogens have been shown to induce effector proteins in the host [42,43,44,45], and the majority of the currently identified resistance gene-encoded proteins commonly contain nucleotide binding (NB) and leucine-rich repeat (LRR) domains [45]. The defense mechanism associated with these proteins is localised cell death [44]. Genes containing NB-LRR domains are common in all plant genomes, while an analysis of the potato genome sequence resulted in the identification of 755 genes that encode polypeptides that contain nucleotide binding (NB) and leucine rich repeat (LRR) domains [46,47]. NB-LRR domains account for the majority of currently identified plant resistance (R) proteins [45], and the NB-LRR, or R genes have been characterised in up to 63 gene clusters within the potato genome [46,47,48]. The resistance mechanisms conferred by qualitative resistance to PVY, either hypersensitive or extreme resistance, are all associated with localised cell death preventing the reproduction of the pathogen, and NB-LRR class genes are viable candidates for a functional role in resistance to this pathogen.

Some studies have identified genes that have been effective against multiple, albeit related, pathogens. The gene *Gro1* provides resistance to all pathotypes of *Globodera rostochiensis* [49,50], while the gene *Ry_sto_* was considered to provide resistance to PVY and PVA [51,52], two distinct viruses, although both are from the genus *Potyvirus* [7]. Identification of genes that provide multiple resistances would be a significant benefit, although these results may represent multiple genes that are closely linked in clusters [2,46]. Although the race-specific resistances may be provided by different genes, gene clusters may still be transferred to progeny in a single block, in the absence of genetic recombination.

These phenotypic screening trials using ELISA to detect virus spread throughout the plant will identify extreme resistance and hypersensitive resistance mechanisms that prevent systemic spread. This is a far superior outcome in terms of plant health and crop hygiene, as multiplication and spread of the pathogen is suppressed. It is also a far superior outcome from a breeding perspective, as resistance mechanisms that prevent the effective pathogen from reproducing will be identified.

For MAS to be of value for increased rates of genetic gain in a plant breeding program, benefits are required in terms of reduced time and/or costs as compared to conventional phenotypic screening. Direct assessment of disease resistance is notoriously error-prone, due to variability of infection rates, and potential changes in population and race structure. Glasshouse-based screening for disease resistance, which could address and mitigate these confounding factors, is costly at an early generation stage due to the high number of genotypes in the breeding population, requiring a large physical space and substantial labour. Screening is usually conducted at a later generation when the number of cultivars is greatly reduced, but this incurs a substantial time delay. A comparison of the cost of glasshouse phenotyping compared to MAS has also shown MAS to be cost-effective [20]. In addition, disease resistance screening can result in an incorrect resistance classification, due to ineffective inoculation or infection. The use of molecular genetic markers in close linkage to resistance genes provides an alternative method with higher accuracy and throughput, and potentially reduced cost and labour. However, for MAS to be effective, the genetic distance between the marker locus and the resistance gene must be minimal, to mitigate the effects of genetic recombination that causes predictive linkages to be lost. The markers in this study exhibited the required diagnostic power for early generation screening, although closely linked, and will require further phenotypic confirmation prior to commercial use to confirm the true phenotypic resistance status.

The identification of cultivars with resistance will enable their use as parents for the development of superior progeny with PVY resistance, and based on the observations reported here, only a limited number of the main Australian commercial cultivars are resistant to PVY. This demonstrates that substantial improvements in the disease resistance of cultivars is possible, providing a viable target for breeding efforts where rapid gains could be made, and as it is cost-effective, the use of MAS would be of great value to create an effective targeted breeding program.

## Figures and Tables

**Table 1:** The resistance response of the main commercial potato cultivars grown in Australia and select cultivars, to two strains of PVY, and the results of the three marker assays.

Cultivar	PVY^O^ ELISA Result	No. of Trials	PVY^O^ R*	PVY^NTN^ ELISA Result	No. of Trials	PVY^NTN^ R*	RYSC3	M45	STM0003
813/28				+	1	S	no	no	no
Almera	-	3	R	+	2	S	no	no	no
Amerosa	+	1	S	+	2	S	no	no	no
Argos				+	2	S	no	no	no
Atlantic	+	6	S	+	7	S	no	no	no
BC0894-2	-	3	R	-	3	R	no	yes	no
Bison	+	1	S	+	3	S	no	no	no
Bliss	+	1	S	+	1	S	no	no	no
Carlingford	-	3	R	-	3	R	no	yes	no
Catani	+	1	S	+	2	S	no	no	no
Charlotte	+	1	S	+	2	S	no	no	no
CMK	+	2	S	+	1	S	no	no	no
Coliban	-	3	R	+	2	S	no	no	no
Crop 17	+	1	S	+	2	S	no	no	no
Crop 8	+	1	S	+	3	S	no	no	no
Crystal	+	1	S	+	1	S	no	no	no
Denali				+	1	S	no	no	no
Desiree	+	1	S	+	2	S	no	no	no
Dutch Cream	+	1	S	+	1	S	no	no	no
Emma	+	1	S	+	3	S	yes	yes	no
Eos	-	3	R	-	3	R	no	yes	no
Eva	-	3	R	-	3	R	yes	yes	no
Exton	-	3	R	+	2	S	no	no	no
FL1867	+	1	S	+	2	S	no	no	no
FL1953	+	1	S	+	2	S	no	no	no
FL2027	+	1	S	+	2	S	no	no	no
Friar	-	3	R	-	3	R	no	yes	no
Galil	-	3	R	-	3	R	no	yes	no
Harmony	+	1	S	+	2	S	no	no	no
Innovator	+	1	S	+	1	S	no	no	no
Kennebec	+	1	S	+	1	S	no	no	no
Kestrel	-	3	R	+	1	S	no	no	no
King Edward	+	1	S	+	1	S	no	no	no
Kipfler	+	1	S	+	1	S	no	no	no
KT3	-	3	R	-	3	R	no	yes	no
Lady Christl	-	4	R	-	4	R	no	yes	no
Lustre	+	1	S	+	1	S	no	no	no
Macrusset	+	1	S	+	1	S	no	no	no
McCain 4	+	1	S	+	1	S	no	no	no
Melody	-	3	R	-	3	R	no	yes	no
Mirridong	+	1	S	+	1	S	no	no	no
Moonlight	+	1	S	+	3	S	no	no	no
Nadine	-	3	R	+	1	S	no	no	no
Nicola	+	1	S	+	1	S	no	no	no
Nooksack	+	1	S	+	1	S	no	no	no
Onka	+	1	S	+	1	S	no	no	no
Otway Red	+	1	S	+	1	S	no	no	no
Pike	+	1	S	+	1	S	no	no	no
Pink Eye	+	1	S	+	1	S	no	no	no
PO3	-	3	R	-	3	R	yes	yes	no
Pontiac	+	2	S	+	1	S	no	no	no
Ranger Russet	+	1	S	+	1	S	no	no	no
Red La Soda	+	1	S	+	1	S	no	no	no
Red Rascal	-	4	R	+	1	S	no	no	no
Rioja	-	3	R	-	3	R	no	no	yes
Riverina Russet	+	1	S	+	1	S	no	no	no
Royal Blue	-	3	R	-	3	R	no	yes	no
Ruby Lou	+	1	S	+	1	S	no	no	no
Russet Burbank	+	7	S	+	5	S	no	no	no
Sebago	+	3	S	+	5	S	no	no	no
Sequoia	-	4	R	+	1	S	no	no	no
Shepody	+	1	S	+	1	S	no	no	no
Simcoe	-	3	R	+	1	S	no	no	no
Snowgem	-	3	R	+	1	S	no	no	no
Sonic	+	2	S	+	1	S	no	no	no
Spunta	+	1	S	+	1	S	no	no	no
Toolangi Delight	+	1	S	+	1	S	no	no	no
Trent	+	2	S	+	1	S	no	no	no
Umatilla	+	2	S	+	1	S	no	no	no
Valor	+	1	S	+	1	S	no	no	no
White Delight	+	1	S	+	2	S	no	no	no
White Lady	-	3	R	-	4	R	no	no	yes
Wilwash	+	1	S	+	1	S	no	no	no
Wontscab	-	3	R	+	2	S	no	no	no

* R = Resistant, S = Susceptible. Note: red shading indicates marker absence with a resistant phenotype, yellow shading indicates marker presence with a susceptible phenotype, and green shading indicates marker presence and a resistant phenotype.

**Table 2:** The Australian resistance classification compared to the resistance ratings listed on the ECPD for PVY and PVY^N^.

Cultivar	Australian Classification PVY^O^ *	Australian Classification PVY^NTN^ *	ECPD Classification PVY **	ECPD Classification PVY^N^ **
Argos		S	v l–l	
Atlantic	S	S	l	h
Charlotte	S	S	m–h	
Crystal	S	S	h	
Denali		S		h–v h
Desiree	S	S	h	h
Eva	R	R	m–h	
Friar	R	R	v h	
Harmony	S	S	l	
Innovator	S	S	m–h	
Kennebec	S	S	m–h	v h
Kestrel	R	S	l–m	
King Edward	S	S	l	
Lady Christl	R	R	v h	v h
Nadine	R	S	h	
Nicola	S	S	m–h	l
Pontiac	S	S	v l–l	
Ranger Russet	S	S	l–m	
Russet Burbank	S	S	l–m	
Sebago	S	S	h	
Shepody	S	S	l–m	v l–l
Spunta	S	S	h	m–h
Umatilla	S	S	l–m	
Valor	S	S	l	
White Lady	R	R	v h	

* Results from this study: R = Resistant, S = Susceptible. ** Resistance rating on the ECPD: l = low, m = moderate, h = high, v = very.

## References

[1] Ahmadvand R., Takacs A., Taller J., Wolf I., Polgar Z. (2012). Potato viruses and resistance genes in potato. Acta Agron. Hung..

[2] Gebhardt C., Valkonen J.P.T. (2001). Organization of genes controlling disease resistance in the potato genome. Ann. Rev. Phytopathol..

[3] Singh R.P., Valkonen J.P.T., Gray S.M., Boonham N., Jones R.A.C., Kerlan C., Schubert J. (2008). Discussion paper: The naming of *Potato virus Y* strains infecting potato. Arch. Virol..

[4] Solomon-Blackburn R.M., Barker H. (2001). A review of host major-gene resistance to potato viruses X, Y, A and V in potato: Genes, genetics and mapped locations. Heredity.

[5] Radcliffe E., Ragsdale D. (2002). Aphid-transmitted potato viruses: The importance of understanding vector biology. Am. J. Potato Res..

[6] Ottoman R., Hane D., Brown C., Yilma S., James S., Mosley A., Crosslin J., Vales M. (2009). Validation and implementation of marker-assisted selection (MAS) for PVY resistance (*Ry_adg_* gene) in a tetraploid potato breeding program. Am. J. Potato Res..

[7] Jefferies C.J. (1998). FAO/IPGRI Technical Guidelines for the Safe Movement of Germplasm, No. 19. Potato. Food and Agriculture Organisation of the United Nations.

[8] Nolte P., Whitworth J.L., Thornton M.K., McIntosh C.S. (2004). Effect of seedborne *Potato virus Y* on performance of Russet Burbank, Russet Norkotah, and Shepody potato. Plant Dis..

[9] Gebhardt C. (2013). Bridging the gap between genome analysis and precision breeding in potato. Trends Genet..

[10] Slater A.T., Cogan N.O.I., Hayes B.J., Schultz L., Dale M.F.B., Bryan G.J., Forster J.W. (2014). Improving breeding efficiency in potato using molecular and quantitative genetics. Theor. Appl. Genet..

[11] Simko I., Jansky S., Stephenson S., Spooner D.M., Vreugdenhil D., Bradshaw J., Gebhardt C., Govers F., MacKerron D.K.L., Taylor M.A., Ross H.A. (2007). Genetics of resistance to pests and diseases. Potato Biology and Biotechnology: Advances and Perspectives.

[12] Bradshaw J.E., Vreugdenhil D., Bradshaw J., Gebhardt C., Govers F., MacKerron D.K.L., Taylor M.A., Ross H.A. (2007). Potato-breeding strategy. Potato Biology and Biotechnology: Advances and Perspectives.

[13] Van Eck H.J., Vreugdenhil D., Bradshaw J., Gebhardt C., Govers F., MacKerron D.K.L., Taylor M.A., Ross H.A. (2007). Genetics of morphological and tuber traits. Potato Biology and Biotechnology: Advances and Perspectives.

[14] Ponz F., Bruening G. (1986). Mechanisms of resistance to plant viruses. Ann. Rev. Phytopath..

[15] Song Y.S., Schwarzfischer A. (2008). Development of STS markers for selection of extreme resistance (*Ry_sto_*) to PVY and maternal pedigree analysis of extremely resistant cultivars. Am. J. Potato Res..

[16] Cockerham G. (1970). Genetical studies on resistance to potato viruses X and Y. Heredity.

[17] Ross H., Parey P. (1986). Potato breeding—Problems and perspectives. Advances in Plant Breeding Verlag.

[18] Valkonen J.P.T., Wiegmann K., Hämäläinen J.H., Marczewski W., Watanabe K.N. (2008). Evidence for utility of the same PCR-based markers for selection of extreme resistance to Potato virus Y controlled by *Ry_sto_* of *Solanum stoloniferum* derived from different sources. Ann. Appl. Biol..

[19] Whitworth J., Novy R., Hall D., Crosslin J., Brown C. (2009). Characterization of broad spectrum potato virus Y resistance in a *Solanum tuberosum* ssp. andigena-derived population and select breeding clones using molecular markers, grafting, and field inoculations. Am. J. Potato Res..

[20] Slater A.T., Cogan N.O.I., Forster J.W. (2013). Cost analysis of the application of marker-assisted selection in potato breeding. Mol. Breed..

[21] Brigneti G., Garcia-Mas J., Baulcombe D.C. (1997). Molecular mapping of the potato virus Y resistance gene *Ry_sto_* in potato. Theor. Appl. Genet..

[22] Flis B., Hennig J., Strzelczyk-Zyta D., Gebhardt C., Marczewski W. (2005). The *Ry-f_sto_* gene from *Solanum stoloniferum* for extreme resistant to Potato virus Y maps to potato chromosome XII and is diagnosed by PCR marker GP122_718_ in PVY resistant potato cultivars. Mol. Breed..

[23] Song Y.-S., Hepting L., Schweizer G., Hartl L., Wenzel G., Schwarzfischer A. (2005). Mapping of extreme resistance to PVY (*Ry_sto_*) on chromosome XII using anther-culture-derived primary dihaploid potato lines. Theor. Appl. Genet..

[24] Witek K., Strzelczyk-Żyta D., Hennig J., Marczewski W. (2006). A multiplex PCR approach to simultaneously genotype potato towards the resistance alleles *Ry-f_sto_* and *Ns*. Mol. Breed..

[25] Cernák I., Taller J., Wolf I., Fehér E., Babinszky G., Alföldi Z., Csanádi G., Polgár Z. (2008). Analysis of the applicability of molecular markers linked to the PVY extreme resistance gene *Ry_sto_*, and the identification of new markers. Acta Biol. Hung..

[26] Van Eck H.J., Vos P.G., Valkonen J.P.T., Uitdewilligen J.G.A.M.L., Lensing H., de Vetten N., Visser R.G.F. (2017). Graphical genotyping as a method to map Ny (o,n)sto and Gpa5 using a reference panel of tetraploid potato cultivars. Theor. Appl. Genet..

[27] Grech-Baran M., Witek K., Szajko K., Witek A.I., Morgiewicz K., Wasilewicz-Flis I., Jakuczun H., Marczewski W., Jones J.D.G., Hennig J. (2020). Extreme resistance to *Potato virus Y* in potato carrying the *Ry_sto_* gene is mediated by a TIR-NLR immune receptor. Plant Biotechnol. J..

[28] Hämäläinen J.H., Watanabe K.N., Valkonen J.P.T., Arihara A., Plaisted R.L., Pehu E., Miller L., Slack S.A. (1997). Mapping and marker-assisted selection for a gene for extreme resistance to potato virus Y. Theor. Appl. Genet..

[29] Hämäläinen J.H., Sorri V.A., Watanabe K.N., Gebhardt C., Valkonen J.P.T. (1998). Molecular examination of a chromosome region that controls resistance to potato Y and A potyviruses in potato. Theor. Appl. Genet..

[30] Sorri V.A., Watanabe K.N., Valkonen J.P.T. (1999). Predicted kinase-3a motif of a resistance gene analogue as a unique marker for virus resistance. Theor. Appl. Genet..

[31] Kasai K., Morikawa Y., Sorri V.A., Valkonen J.P., Gebhardt C., Watanabe K.N. (2000). Development of SCAR markers to the PVY resistance gene *Ry_adg_* based on a common feature of plant disease resistance genes. Genome.

[32] Dalla Rizza M., Vilaró F., Torres D., Maeso D. (2006). Detection of PVY extreme resistance genes in potato germplasm from the Uruguayan breeding program. Am. J. Potato Res..

[33] Herrera M.D.R., Vidalon L.J., Montenegro J.D., Riccio C., Guzman F., Bartolini I., Ghislain M. (2018). Molecular and genetic characterization of the Ry adg locus on chromosome XI from Andigena potatoes conferring extreme resistance to potato virus Y. Theor. Appl. Genet..

[34] Clark M.F., Adams A.N. (1977). Characteristics of the microplate method of enzyme-linked immunosorbent assay for the detection of plant viruses. J. Gen. Virol..

[35] Schultz L., Cogan N.O.I., McLean K., Dale M.F.B., Bryan G.J., Forster J.W., Slater A.T. (2012). Evaluation and implementation of a potential diagnostic molecular marker for *H1*-conferred potato cyst nematode resistance in potato (*Solanum tuberosum* L.). Plant Breed..

[36] Milbourne D., Meyer R.C., Collins A.J., Ramsay L.D., Gebhardt C., Waugh R. (1998). Isolation, characterisation and mapping of simple sequence repeat loci in potato. Mol. Gen. Genet..

[37] Kehoe M., Jones R. (2011). A proposal to help resolve the disagreement between naming of potato virus Y strain groups defined by resistance phenotypes and those defined by sequencing. Arch. Virol..

[38] Heldák J., Bežo M., Štefúnová V., Galliková A. (2007). Selection of DNA markers for detection of extreme resistance to potato virus Y in tetraploid potato (*Solanum tuberosum* L.) F1 progenies. Czech J. Genet. Plant Breed..

[39] Jones R.A.C. (1990). Strain group specific and virus specific hypersensitive reactions to infection with potyviruses in potato cultivars. Ann. Appl. Biol..

[40] Valkonen J.P.T., Slack S.A., Plaisted R.L. (1994). Use of the virus strain group concept to characterize the resistance to PVX and PVYo in the potato cv Allegany. Am. Potato J..

[41] Solomon-Blackburn R.M., Bradshaw J.E. (2007). Resistance to potato virus Y in a multitrait potato breeding scheme without direct selection in each generation. Potato Res..

[42] Birch P.R.J., Rehmany A.P., Pritchard L., Kamoun S., Beynon J.L. (2006). Trafficking arms: Oomycete effectors enter host plant cells. Trends Microbiol..

[43] Catanzariti A.-M., Dodds P.N., Ellis J.G. (2007). Avirulence proteins from haustoria-forming pathogens. FEMS Microbiol. Lett..

[44] Jones J.D.G., Dangl J.L. (2006). The plant immune system. Nature.

[45] Tameling W., Takken F. (2008). Resistance proteins: Scouts of the plant innate immune system. Eur. J. Plant Pathol..

[46] Bakker E., Borm T., Prins P., van der Vossen E., Uenk G., Arens M., de Boer J., van Eck H., Muskens M.L., Vossen J. (2011). A genome-wide genetic map of NB-LRR disease resistance loci in potato. Theor. Appl. Genet..

[47] Jupe F., Witek K., Verwejj W., Sliwka J., Pritchard L., Etherington G.J., Maclean D., Cock P.J., Leggett R.M., Bryan G.J. (2013). Resistance gene enrichment sequencing (RenSeq) enables reannotation of the *NB-LRR* gene family from sequenced plant genomes and rapid mapping of resistance loci in segregating populations. Plant J..

[48] Jupe F., Pritchard L., Etherington G.J., MacKenzie K., Cock P.J.A., Wright F., Sharma S.K., Bolser D., Bryan G.J., Jones J.D.G. (2012). Identification and localisation of the *NB-LRR* gene family within the potato genome. BMC Genom..

[49] Barone A., Ritter E., Schachtschabel U., Debener T., Salamini F., Gebhardt C. (1990). Localization by restriction fragment length polymorphism mapping in potato of a major dominant gene conferring resistance to the potato cyst nematode *Globodera rostochiensis*. Mol. Gen. Genet..

[50] Gebhardt C., Bellin D., Henselewski H., Lehmann W., Schwarzfischer J., Valkonen J. (2006). Marker-assisted combination of major genes for pathogen resistance in potato. Theor. Appl. Genet..

[51] Barker H. (1996). Inheritance of resistance to potato viruses Y and A in progeny obtained from potato cultivars containing gene *Ry*: Evidence for a new gene for extreme resistance to PVA. Theor. Appl. Genet..

[52] Barker H. (1997). Extreme resistance to potato virus V in clones of *Solanum tuberosum* that are also resistant to potato viruses Y and A: Evidence for a locus conferring broad-spectrum potyvirus resistance. Theor. Appl. Genet..

